# Optical Sensing of the Fatigue Damage State of CFRP under Realistic Aeronautical Load Sequences

**DOI:** 10.3390/s150305710

**Published:** 2015-03-09

**Authors:** Pablo Zuluaga-Ramírez, Álvaro Arconada, Malte Frövel, Tomás Belenguer, Félix Salazar

**Affiliations:** 1 Instituto Nacional de Técnica Aeroespacial (INTA), Carretera de Ajalvir Km 4, 28850 Torrejón de Ardoz, Spain; E-Mails: frovelm@inta.es (M.F.); belenguer@inta.es (T.B.); 2 Department of Applied Physics, ETSI Minas, Universidad Politécnica de Madrid, C/Ríos Rosas 21, 28003 Madrid, Spain; E-Mails: a.arconada@me.com (Á.A.); felixjose.salazar@upm.es (F.S.)

**Keywords:** composite structures, surface roughness, fatigue damage, variable amplitude loads, optical inspection

## Abstract

We present an optical sensing methodology to estimate the fatigue damage state of structures made of carbon fiber reinforced polymer (CFRP), by measuring variations on the surface roughness. Variable amplitude loads (VAL), which represent realistic loads during aeronautical missions of fighter aircraft (FALSTAFF) have been applied to coupons until failure. Stiffness degradation and surface roughness variations have been measured during the life of the coupons obtaining a Pearson correlation of 0.75 between both variables. The data were compared with a previous study for Constant Amplitude Load (CAL) obtaining similar results. Conclusions suggest that the surface roughness measured in strategic zones is a useful technique for structural health monitoring of CFRP structures, and that it is independent of the type of load applied. Surface roughness can be measured in the field by optical techniques such as speckle, confocal perfilometers and interferometry, among others.

## 1. Introduction

Carbon fiber reinforced polymers (CFRPs) are widely used and their industrial applications have been growing continuously for the last decades. The aerospace industry often uses composite materials (mainly CFRP) in critical components or subsystems subjected to cyclic loads during the service life that generate accumulation of damage due to fatigue [1]. Quantifying and monitoring the accumulated fatigue damage is essential to predict the remaining life of a component or structure, and optimize intervals of inspection, repair and replacement.

The most used technique to evaluate the damage state due to fatigue loads in CFRP structures, is to measure the structural loads during the service life using electrical strain gauge sensors. These types of sensors are not completely reliable because they are altered by environmental conditions and by fatigue loads. The strain gauge measurements are also affected by the changes of the CFRP stiffness due to the damage caused by the fatigue loads, which leads to a different stress-strain relation and hence an incorrect value of the measured loads.

Another disadvantage of measuring the service loads of CFRP structures is that the accumulated fatigue damage is conventionally calculated using linear models such as Palmgren-Miner, developed for homogeneous materials [2], and the predictions are not reliable [3]. Experimental studies show that life predictions for composite materials under VALs, calculated by the Palmgren-Miner rule, produce non-conservative and inaccurate results [4,5,6].

To avoid the aforementioned disadvantages, alternative techniques have been developed to determine the fatigue damage state. These are typically based on measuring material properties such as the stiffness degradation of the material [7,8,9]. Other techniques are based on phenomenological changes [2] such as ultrasonic methods [10], acoustic emissions [11], infrared imaging, thermography [12,13], electrical resistance [14], digital image correlation [13,15], and X-ray tomography [16].

**Figure 1:**
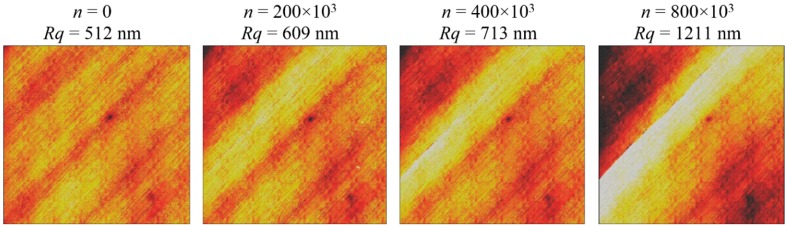
Evolution of the surface topography due to fatigue damage of a CFRP specimen.

Previous studies [17,18] have shown that the magnitude of the surface roughness increases due to changes in the topography that have been induced by the damage. The changes of the surface start at the beginning of the life of the structure with matrix micro-cracks. With increasing cycles, the cracks become bigger and produce local delaminations. At the end of the life the local delaminations become deeper. Figure 1 shows the evolution of the surface topography for a particular and representative case of a CFRP specimen after different numbers of fatigue cycles. From previous studies we have established that the roughness parameter which has a better correlation with the accumulated damage of the material is the root mean square (standard deviation), commonly known as *Rq*, which is determined by the next equation, where *z_i_* is the value of the height of the measured point *i* respect to the mean plane of the surface, and *P* is the total number of measured points (pixels) per surface [19,20]:

(1) $$Rq=\;\sqrt{\frac{1}{P}\overset{P}{\underset{i=1}{\sum }}z_{i}^{2}}$$

Visual inspections of the specimen’s lateral cross section of the composite laminate show that the internal and the surface layers have the same damage characteristics [17]. This observation suggests that inspections of the surface layer can be extrapolated to the whole laminate and could give an idea of the damage state of the entire component or structure. In a second work done by the authors, this hypothesis was validated by comparing the surface roughness of specimens cycled at constant amplitude load (CAL) with respect to the stiffness degradation obtaining a good correlation coefficient [18].

There are different techniques for measuring roughness [19]. One of the most employed is the mechanical profilometer. This apparatus allows the determination of the surface heights by means of a stylus coupled with a displacement sensor. The data obtained along parallel scanning lines may be processed giving information of important roughness parameters about the magnitude and statistics. This technique is very precise, however it needs measuring the region of study line by line, which leads to a considerably consumption of time. Another drawback of this method is the impossibility to employ it on soft surfaces like some plastics and rubbers, in which the direct contact between the needle and the sample can damage the surface, thus giving false measurement data. On the contrary contact-free methods for characterizing surface roughness have some advantages. In fact, they do not need the contact between the specimen to be studied and the measuring system; they are fast and also precise. Among them, optical techniques are going to be very used due to their advantages. Specifically, speckle techniques and confocal microscopy are two of the most useful methods [21,22], which may be employed with a reasonable economic cost and short time when comparing them with other methods [12,13,16].

In this article we have used a confocal microscope for determining the roughness of all the samples chosen. By this method each point of the rough surface is illuminated by a source and the collection system is focused at the same time and on the same point. The specimen is scanned vertically in many steps, so that every point on the surface passes through a pinhole to the focus plane. The surface height at each pixel location on the CCD system is formed by picking up the peak of the narrow axial response. After this process, a micro-surface image is obtained by the addition of the information of all the different acquisition planes during the scanning process.

The present study is focused on evaluating the evolution of the surface roughness due to realistic fatigue loads, such as the typically most critical structural loads supported by the wing root of a fighter aircraft, during different types of missions and maneuvers. These loads are represented by the FALSTAFF standardized load sequence [23]. As mentioned by Sonsino [3], variable amplitude load tests are necessary to understand the behavior of the material under real in-service conditions, due to the absence of effective cumulative damage models that predicts the life of components under VAL using CAL test data.

## 2. Materials and Methodology 

### 2.1. Materials and Test Set up

The material selected to evaluate the methodology is a relatively new CFRP with an epoxy matrix (MTM-45-1/IM7) from Cytec Industires Inc. (Woodland Park, NJ, USA) used for aeronautic structures. One panel of 2 mm thickness has been manufactured with a quasi-isotropic stacking sequence of ((45,90,−45,0)_s_)_2_ and has been autoclave cured at 6 bars and 130 °C. Its ultimate tensile strength (*S_ut_*) of 938 MPa was statistically determined in a previous study [17]

**Figure 2:**
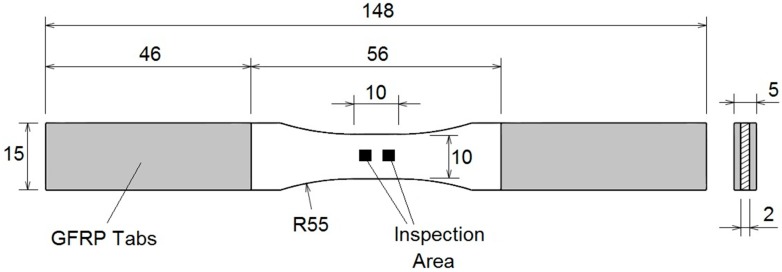
“Dog bone” geometry of the CFRP coupons used for tensile variable amplitude tests. Inspection area of one of the faces of the coupon.

Nine coupons with “dog bone” geometry have been manufactured from the panel (see Figure 2). This geometry is selected to guarantee that the damage of the coupon is produced in a controlled zone, (gage zone) which corresponds to a volume of 10 × 10 × 2 mm^3^. Tabs made of GFRP were bonded on the specimen ends in order to avoid spurious stresses due to the gripping force of the test machine clamps.

**Figure 3:**
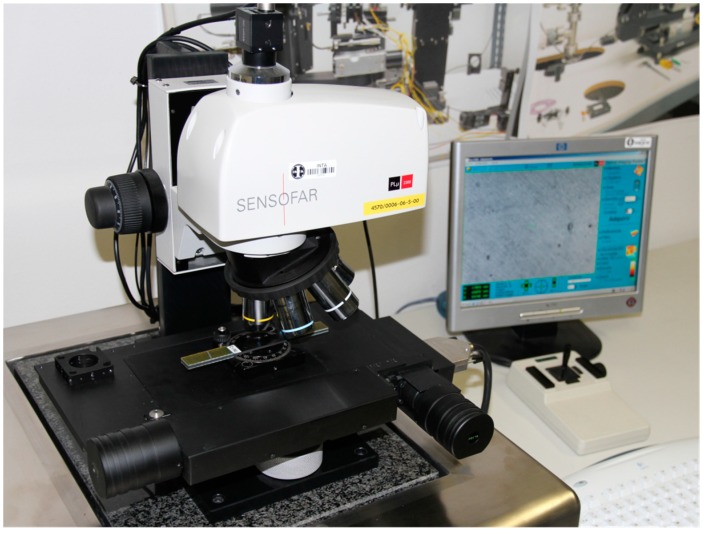
Experimental setup to measure the surface roughness by the confocal microscope.

A hydraulic test machine (MTS 810 from MTS Systems Corporation, Eden Prairie, MN, USA), under load controlled conditions, was used for the fatigue tests. The surface topography was obtained by a confocal microscope (PLμ Confocal Imaging Profiler, from Sensofar, Barcelona, Spain) with an objective zoom of 50× and an axial resolution of 5 nm. The experimental setup to measure the surface roughness of the coupons with the confocal microscope is shown in the Figure 3.

### 2.2. Fatigue Tests with Variable Amplitude Loads

The FALSTAFF load sequence is a standardized representation of the loads supported by the wing roots of fighter aircrafts during 200 flights (1 year of typical usage), under the combinations of different types of missions and maneuvers [23]. The total number of cycles of the FALSTAFF load sequence is 1.054 × 10^6^ cycles of different magnitude, and has been applied to the coupons in order to evaluate the fatigue damage under VAL.

**Figure 4:**
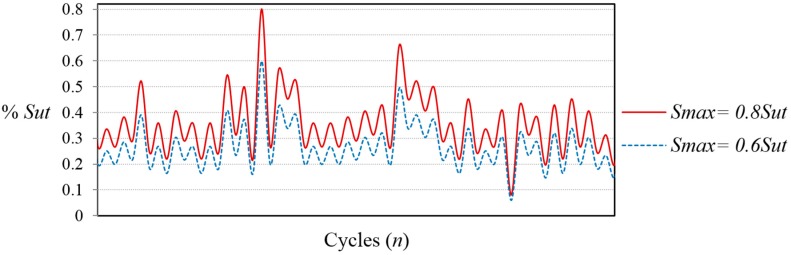
Fragment of the FALSTAFF load sequence with two different magnitudes of maximum peak load.

VALs have been applied at 10 Hz with an R = 0.1, where the relation of the maximum peak load of the sequence and the minimum valley of the sequence is 10:1. In order to evaluate load sequences of different severity, FALSTAFF has been scaled to different magnitudes of maximum peak load. Table 1 shows the load sequences applied to the coupons and Figure 4 illustrates a fragment of the load history applied to the coupons with two different magnitudes of maximum peak load. If the coupon did not fail within the load history, the sequence was repeated until failure, with a limit of 10 million of cycles. The fatigue tests have been interrupted periodically before failure in order to perform the measurement of the surface parameters.

**Table 1:** Different magnitude of load sequences applied to the evaluated coupons.

Coupon	Max Peak (*%Sut*)	Min Valley (*%Sut*)
C01	80%	8%
C02	80%	8%
C03	75%	8%
C04	75%	8%
C05	70%	7%
C06	70%	7%
C07	65%	7%
C08	65%	7%
C09	60%	6%

### 2.3. Surface Inspection by Confocal Microscope

Measuring the surface roughness in several points is recommended in order to obtain an adequate statistic of the damage. In the present work, the surface roughness of each coupon was measured in four points in the gage zone, two points in the front face and two points in the back face (see Figure 2). Each test point has an area of 1.55 × 1.49 mm^2^, which has been established in order to evaluate a similar area and scale than the used in laser speckle techniques. The topography of the surface at each area of evaluation is obtained by confocal microscope by measuring the heights of the surface in a discretized area of 4675 × 4478 pixels, where each pixel has an area of 0.33 × 0.33 µm^2^. To present the results of roughness evolution, the four points of examination have been averaged with the aim to present one value of evolution per coupon.

## 3. Results and Discussion

### 3.1. Gassner Curve and Stiffness Degradation

**Figure 5:**
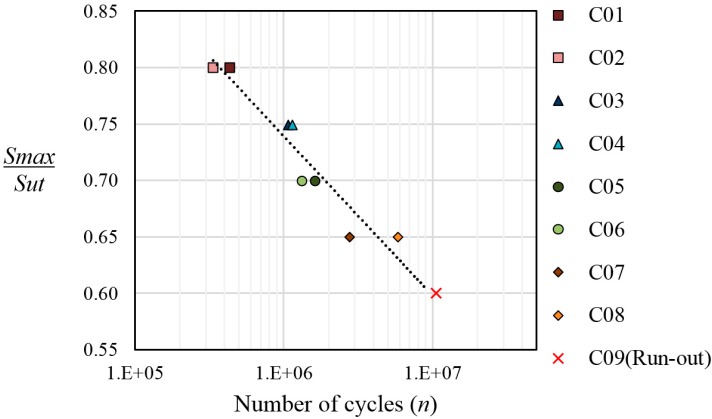
Gassner curve in semi-log graphic, for the coupons cycled with the FALSTAFF load sequence at different levels of maximum peak load.

The classical stress-life curve (S-N) which is used to characterize the fatigue properties at CALs, can be used in a modified version for VALs to present life results, by plotting the maximum peak stress (*S_max_*) *versus* the number of cycles until failure (N). The resultant curve is known as Gassner curve [3]. Figure 5 shows the Gassner curve for the coupons cycled at different levels of maximum peak load. The trend line shown in the graphic has been adjusted using the failure data of the coupons C01 to C08. The coupon C09 loaded at *S_max_ =* 0.6*S_ut_* survives 10.5 × 10^6^ cycles and its failure cycle is unknown.

**Figure 6:**
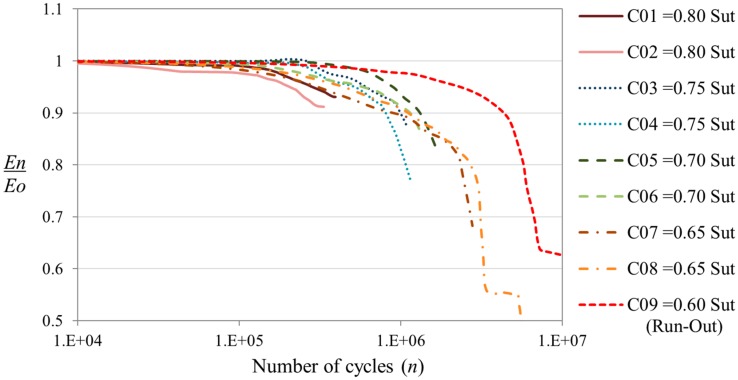
Stiffness degradation in semi-log graphic, for the coupons cycled with the FALSTAFF load sequence at different levels of maximum peak load.

The stiffness degradation of the coupons has been measured during the fatigue cycles. The value of the stiffness has been calculated taking into account the displacement of the test machine and the applied load. The stiffness measurements have been treated in order to obtain normalized values and to quantify the stiffness degradation in terms of percentage of the initial stiffness. Figure 6 shows the degradation of the stiffness due to the accumulated damage of the coupon. The plot shows that the coupons cycled with VALs of higher severity presents a faster degradation of the stiffness, because higher loads produce more damage in the composite material. This behavior was expected and is in accordance with the results obtained when CALs are applied [18] and confirms that the stiffness degradation can be used as a metric to determine the damage state due to fatigue loads.

### 3.2. Surface Roughness Evolution through Cycles

**Figure 7:**
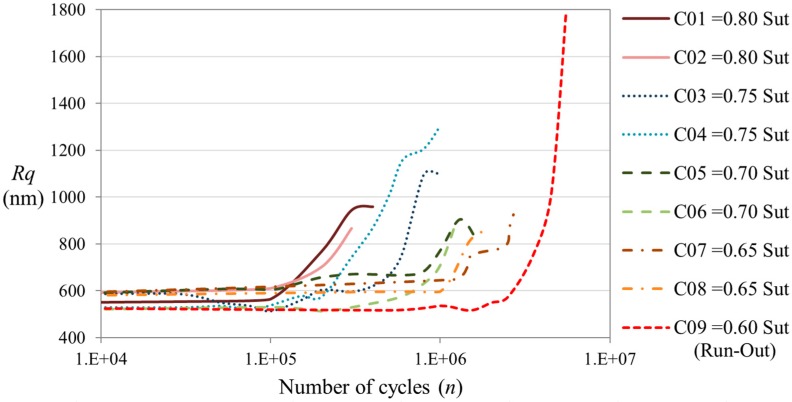
Evolution of the surface roughness magnitude quantified by the parameter *Rq* during the VALs.

Figure 7 shows the evolution of the surface roughness magnitude due to damage accumulation in the material. The magnitude of the roughness has been quantified by the parameter *Rq*, Equation (1). The graphic shows that the magnitude of the roughness increases due to the accumulation of damage on the material. The rate of increment is highly dependent on the applied load level. The roughness grows faster when the applied load spectra is more severe. This is because a faster growing of the damage is produced in the material when is cycled at higher loads. This behavior is similar to the results of stiffness degradation.

### 3.3. Surface Roughness versus Stiffness Degradation

**Figure 8:**
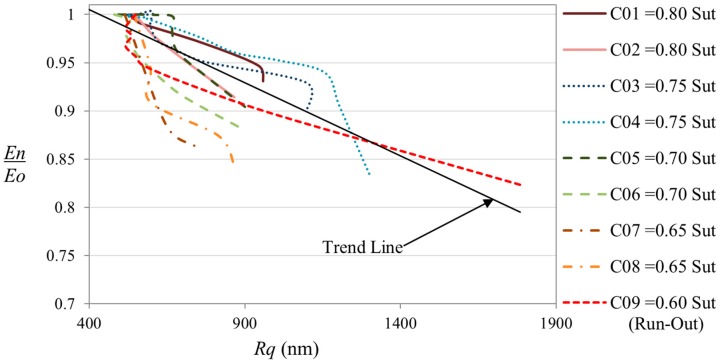
Correlation between stiffness degradation and surface roughness magnitude (*Rq*) at VALs. The trend line is obtained combining the roughness and stiffness data of all the coupons.

Figure 8 shows the relation between the structural damage measured by the roughness magnitude *Rq* and the damage measured by the stiffness degradation. The graphic shows that the surface roughness tends to increase with the degradation of the stiffness. A linear Pearson correlation between both parameters has been determined with a magnitude of 0.75. We consider this correlation as good for the case of evaluate fatigue damage by non-destructive methods. This is because the fatigue is a stochastic phenomenon. The scattering in the life results for the evaluated material when the same geometry and the same level of load is applied, could be around one decade in the logarithmic scale [6]. This scattering is due to manufacturing process of the CFRP that could produce different type of imperfections in each coupon.

### 3.4. Comparison between Results Obtained in Previous Studies for CALs and the Results Obtained for VALs

**Figure 9:**
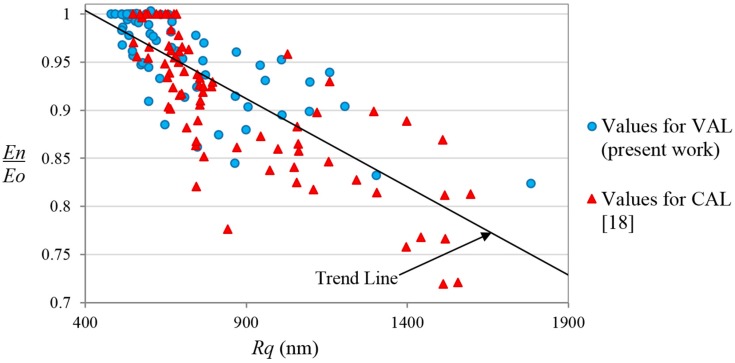
Comparison between the data obtained for VAL in the present work, and the data obtained for CAL in a previous study [18]. The trend line is obtained for the combination of both studies.

The results of the correlation between the stiffness degradation and the increment of the roughness magnitude, obtained in the present work with realistic in-service loads (VAL), have been compared with results reported in previous studies obtained with the same methodology for CAL [18]. Figure 9 shows both data superimposed. The results for both studies show similar tendencies and similar statistics and scattering. Taking into account both data (CAL and VAL), the Pearson correlation coefficient between stiffness degradation and roughness magnitude is 0.80. We can conclude from this comparison that the results obtained by the present methodology are independent of the load type applied, and can be used for applications where the in-service loads are known and controlled (such as pressure vessels) and for applications where the loads are of random or variable nature (wings, automotive components, *etc.*).

## 4. Conclusions

There is a growing demand for evaluating the fatigue state of in-service structures and components made of composite materials. The non-destructive methodologies employed to assess the fatigue damage state of composite materials are still in development. The present work presents the evaluation of a new optical sensing methodology to determine the fatigue damage state of CFRP cycled with realistic in-service loads. The in-service loads have been applied following the standard load sequence for fighter aircraft (FALSTAFF).

The results show that the fatigue loads produce significant changes of the surface topography that increases the magnitude of the surface roughness. The increment on the surface roughness has been correlated with the internal damage of the material by comparing the measurements with the stiffness degradation obtaining a linear relation, with a Pearson correlation coefficient of 0.75.

The results obtained by the present work under realistic in-service loads have been compared with results obtained in a previous work for constant amplitude loads obtaining similar tendencies and statistics. Taking into account both data (CAL and VAL), the Pearson correlation coefficient between stiffness degradation and roughness magnitude is 0.80. We can conclude from this comparison that the results obtained with the present methodology are independent on the load type applied, and can be used for applications where the in-service loads are known and controlled (such as pressure vessels) and for applications where the loads are of random or variable nature (wings, automotive components, *etc.*).

## Nomenclature

CFRP = Carbon Fiber Reinforced Polymer
GFRP = Glass Fiber Reinforced Polymer
VAL = Variable Amplitude Load
CAL = Constant Amplitude Load
*S_ut_* = Ultimate Tensile Strength
*S_max_* = Maximum peak stress during the load history
FALSTAFF = Fighter Aircraft Loading STAndard for Fatigue
*n* = Number of cycles
N = Number of cycles at failure also known as life.
R = Stress Ratio (*S_min_*/*S_max_*)
*Rq* = Roughness parameter which represents the root mean square of the heights
*P =* Total number of pixels measured in an area of evaluation
*z_i_ =* height of the measured point “*i”* respect to the mean plane of the surface
*En/Eo* = normalized stiffness degradation at cycle *n*
*Eo* = Initial stiffness
*En =* Stiffness at cycle *n*
S-N = Stress vs Life curve
